# Aphids on Aphid-Susceptible Cultivars Have Easy Access to Turnip Mosaic Virus, and Effective Inoculation on Aphid-Resistant Cultivars of Oilseed Rape (*Brassica napus*)

**DOI:** 10.3390/plants12101972

**Published:** 2023-05-13

**Authors:** Zhong-Ping Hao, Zeng-Bei Feng, Lei Sheng, Wei-Xin Fei, Shu-Min Hou

**Affiliations:** Crop Research Institute, Anhui Academy of Agricultural Sciences, Hefei 230031, China; hzp5187@sina.com (Z.-P.H.); zaoanxun@163.com (Z.-B.F.); 13685514362@163.com (L.S.); fei_weixin@163.com (W.-X.F.)

**Keywords:** cabbage aphid, electropenetrography, oilseed rape, probing behavior, transmissibility, turnip mosaic virus, vector

## Abstract

Plant viruses improve transmission efficiency by directly and indirectly influencing vector behavior, but the impact of plant cultivars on these modifications is rarely studied. Using electropenetrography (EPG) technology, a comparative study of the effects of turnip mosaic virus (TuMV) infection on quantitative probing behaviors of the cabbage aphid (*Brevicoryne brassicae*) was conducted on two oilseed rape cultivars (‘Deleyou6’ and ‘Zhongshuang11’). Compared to mock-inoculated plants, cabbage aphids on infected plants increased the frequency of brief probing, cell penetration, and salivation. Additionally, aphids on infected ‘Deleyou6’ prolonged cell penetration time and decreased ingestion, but not on infected ‘Zhongshuang11’, suggesting that aphids were more likely to acquire and vector TuMV on the aphid-susceptible cultivar ‘Deleyou6’ than on resistant cultivars. TuMV also affected aphid probing behavior directly. Viruliferous aphids reduced the pathway duration, secreted more saliva, and ingested less sap than non-viruliferous aphids. In comparison with non-viruliferous aphids, viruliferous aphids started the first probe earlier and increased brief probing and cell penetration frequencies on the aphid-resistant cultivar ‘Zhongshuang11’. Based on these observations, viruliferous aphids can be inoculated with TuMV more efficiently on ‘Zhongshuang11’ than on ‘Deleyou6’. Although aphid resistance and TuMV infection may influence aphid probing behavior, oilseed rape resistance to aphids does not impede TuMV transmission effectively.

## 1. Introduction

Most phytoviruses are transmitted to hosts by insect vectors [1]. Three events need to be taken into account for phytovirus transmission: (1) acquisition during the vector ingestion on an infected plant; (2) retention or circulation within the vector organism; and (3) inoculation during new vector ingestion on a healthy plant [2]. In the process of virus transmission, plant viruses can impact their vectors’ behavior and fitness both directly (via the presence of the virus in the vector’s body) and indirectly (by changes in the physiological and biochemical properties of the plant as a result of infection) to facilitate their spread [3,4].

Phytoviruses that are non-persistently and non-circulatively transmitted are only found at the vector’s stylet tips [2]. These viruses are acquired and transmitted by the insect vectors during brief probing punctures in plant epidermal and mesophyll cells [2] and tend to have temporary or context-specific associations with their vectors, which can have a variety of impacts, from increased attraction to infected plants, quick dispersal to preferred settlement and arrestment on infected plants, and improved fitness [5].

Non-persistent and non-circulative phytoviruses indirectly influence their vectors through shared host plants [2]. These viruses make plants more attractive, especially through visual and olfactory features. For example, upon enhancing the concentration of the attraction chemicals (Z)-3-hexenyl acetate on infected red raspberry (*Rubus idaeus* Linnaeus), black raspberry necrosis virus (BRNV) and raspberry leaf mottle virus (RLMV) manipulated the behavior of the raspberry aphid, *Amphorophora idaei* Börner (Hemiptera: Aphididae) [6]. Well-developed symptoms of pea enation mosaic virus induce a strong preference for *Acyrthosiphon pisum* Harris (Hemiptera: Aphididae), probably in response to visual cues [7].

Non-persistent and non-circulative phytoviruses, on the other hand, reduce plant suitability for their vectors in order to encourage the vectors to visit other plants to enable them to spread. They reduce or at least do not increase plant palatability to deter their vectors from sustained feeding due to the possibility of losing their infectiousness [8]. For example, cucumber mosaic virus (CMV) infection caused the biosynthesis of 4-methoxy-indol-3-yl-methylglucosinolate in *Arabidopsis thaliana* (L.) Heynh, which prevented *Myzus persicae* Sulzer (Hemiptera: Aphididae) from phloem ingestion [9]. Additionally, numerous studies have demonstrated that non-persistent and non-circulative phytovirus-infected plants have less favorable aphid settling preferences and life history traits (for review: see Ref. [2]).

For non-persistent viruses, CMV provides a thorough illustration of the impact of viral infection on aphid-feeding behavior. CMV has been reported to induce specific biochemical alterations in plant hosts that alter the alighting, settling, and probing behaviors as well as the fitness of its vectors *Aphis gossypii* Glover (Hemiptera: Aphididae) and *M. persicae* [4,10]. The biochemical changes in host plants include reduced host-plant quality, such as decreased carbohydrates and amino acids in leaf tissue and phloem, and adjustments in plant stress hormones, leading to rapid vector dispersal [11]. However, in some pathosystems, the opposite is true. Potato virus Y, Turnip mosaic virus (TuMV), and Zucchini yellow mosaic virus have been reported to counter plant host defenses and/or enrich host plant nutritional quality for aphids (for review: see Ref. [5]). Thus, there are two strategies that phytoviruses use to promote their transmission: (1) the ‘quick dispersion-low nutrition’ strategy, which relies on rapid attraction and dispersal of aphids from less-nutritious infected plants, and (2) the ‘slow dispersion-high nutrition’ strategy, which relies on increased arrest on highly-nutritious infected plants facilitating rapid wing development and subsequently virus spread [11].

A virus’ ability to modify vector-feeding behavior directly to facilitate its transmission is highly adaptive [3,12,13,14]. For non-persistently transmitted viruses, the direct effects of these viruses on vectors are less well studied. However, specific interactions occur, rather than infecting aphid-stylets with virions, such as with cauliflower mosaic virus (CaMV). Three CaMV-encoded proteins interact in order for CaMV to transmit to aphid [15]. One of these proteins, CaMV-encoded P2, is a non-virion helper component protein, which via its N-terminus, attaches to the aphid-stylet, and its C-terminus binds to P3’s N-terminal region, which anchors inside the virion capsid shell [16,17]. As aphids salivate while probing, the saliva runs through the acrostyle region, which is the most likely a way to inoculate plants [1]. As a result of the fact that CaMV virions are found in the vector aphid acrostyle, which is a particular stylet region that contains unique non-glycosylated proteinaceous receptors [18]. The TuMV transmissible complex is composed of the filamentous virus particle and the helper component (helper component protein) HC-Pro. HC-Pro is a 52 kDa multifunctional protein involved not only in vector transmission but also in the suppression of gene silencing and other plant defense reactions, in viral movement, and in the processing of the viral precursor polyprotein [19]. Usually, compared with non-viruliferous vectors, viruliferous vectors were less or not responsive to volatile organic compounds. The viruses may play a direct regulatory role in the olfactory response of the vectors [20].

As a non-persistently and non-circulatively transmitted virus, TuMV can infect the oilseed rape *Brassica napus* L. [21,22], resulting in a yield reduction of up to 70% [22]. TuMV was found in more than 24% of the cases of oilseed rape diseases in ten different regions of China, resulting in a 50 to 80% yield loss in oilseed rape [21]. The main TuMV vectors in significant rapeseed-producing regions along the lower reaches of the Yangtze River in China were *Lipaphis erysimi* Kaltenbach and *M. persicae*, but *Brevicoryne brassicae* L. (Hemiptera: Aphididae) population has steadily grown and received more attention lately than the other two species [23,24]. An increase in viral symptoms (leaf mosaic, vein clearing, leaf and stem distortion, and growth loss) has been noted in several rapeseed-producing regions when a high prevalence of *B. brassicae* has been found (Investigation reports from China Agriculture Research System, unpublished reports). The traditional prevention effect of chemical pesticides is only temporary, which can cause significant environmental harm. As a result, the cultivation and promotion of resistance cultivars are one of the most cost-effective and effective measures for preventing and controlling TuMV. Further elucidating the interactions of the three species (oilseed rape–TuMV–cabbage aphid) could not only aid in understanding the mechanism of species formation and constructing the co-evolution model among insects, TuMV, and plants but also in coordinating the relationship between plant resistance and biological control in production, which would provide theoretical guidance for opening up new methods for aphid and TuMV management [25].

EPG can track and quantify invisible stylet-probing activities in real-time, which could reflect plant pathogen transmission behaviors and assist researchers in better understanding plant resistance mechanisms [26]. By analyzing EPG waveforms that have been characterized and defined, the hindering properties of different tissue levels on oilseed rape leave against aphids could be assessed [27,28]. Several studies based on the analysis of EPG variables have noted the alteration of aphid feeding behavior associated with the infectious state of the insect and/or the host plant that may influence the phytovirus spread [2]. Using EPGs, in the current study, we assessed the effects of three treatments on two cultivars of oilseed rape. In addition, we analyzed the effects of aphid resistance to oilseed rape on TuMV transmission by aphids.

## 2. Results

### 2.1. Aphid and TuMV Incidence in the Filed

**Figure 1:**
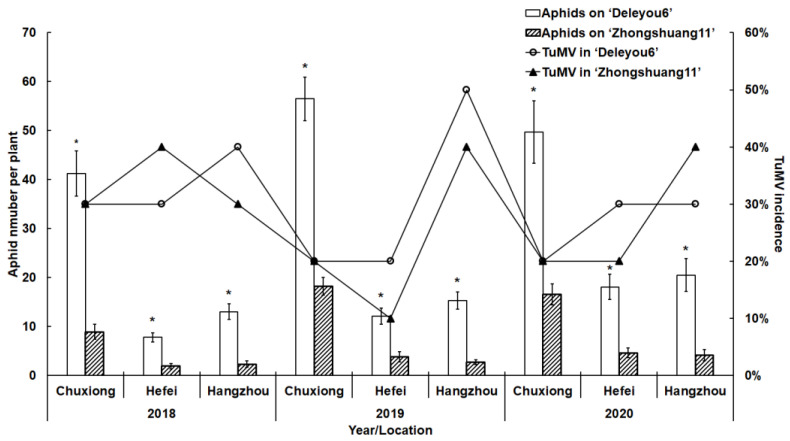
The number of aphids per plant and TuMV incidence on the two oilseed rape cultivars at three locations surveyed for three years. The column graph represents the number of aphids per plant and the line graph represents the incidence of TuMV. The figure’s values display mean ± standard error (SE) (for aphid number per plant) and percentage (for TuMV incidence). These data were compared using Student’s *t*-tests (for Gaussian variables) or Mann–Whitney *U*-tests (for non-Gaussian variables). The significance level was chosen at *p* < 0.05. The * on the white columns denotes a statistical difference between the two oilseed rape cultivars. Chuxiong in Yunnan province (25°19′12″ N, 101°29′24″ E), Hefei in Anhui province (31°52′48″ N, 117°14′24.00″ E), Hangzhou in Zhejiang province (30°18′36″ N, 120°11′24″ E).

The results of the three-year survey showed that aphids were significantly more numerous on ‘Deleyou6’ than on ‘Zhongshuang11’ at all three locations (Chuxiong, Hefei, and Hangzhou). Over three years, averaging around 30%, there was no discernible difference in the incidence of TuMV between ‘Deleyou6’ and ‘Zhongshuang11’ at the three locations (U = 37.00, *p* = 0.7949, Figure 1).

### 2.2. Identification of TuMV-Resistance of Plants

The average TuMV incidence between the two cultivars was not significantly different. Although the average disease index of ‘Deleyou6’ was significantly higher than that of ‘Zhongshuang11’ (F = 1.09, *p* = 0.0106), the TuMV-resistance levels of the two cultivars were evaluated as medium resistance (Table 1).

### 2.3. Identification of Aphid-Resistance of Plants

**Figure 2:**
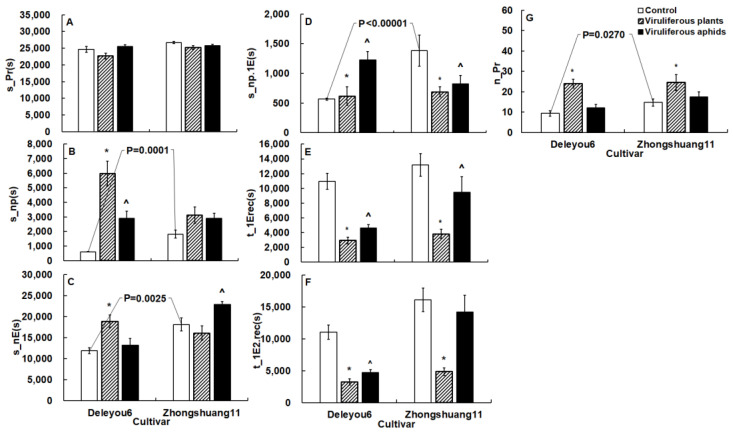
The overview variables of *Brevicoryne brassicae* probing behavior on the two oilseed rape cultivars in the TuMV-infected and mock-inoculated treatments. The figure’s values display mean ± standard error (SE). Following the square-root transformation for frequency variables, natural log transformation for time variables, and square-arcsine for percentage variables, these data were compared using Student’s *t*-tests (for Gaussian variables) or Mann–Whitney *U*-tests (for non-Gaussian variables). It was decided to set the significance level at *p* < 0.05. The *p*-value on the white columns indicates a statistical difference between the two cultivars; the * on the gray columns denotes a statistical difference between infected plant and mock-inoculated control in the same cultivar; the ^ on the black columns shows a statistical difference between viruliferous aphids and non-viruliferous aphids in the same cultivar. (**A**): Total probing time; (**B**): Total time of the non-probing intervals; (**C**): Total duration of the no phloematic phase; (**D**): Duration of the nonprobe period before the first E; (**E**): Time from the start of EPG to the first E; (**F**): Time from the start of EPG to the first E2; (**G**): Number of probes. The acronyms for the variables are defined in Table S1.

**Figure 3:**
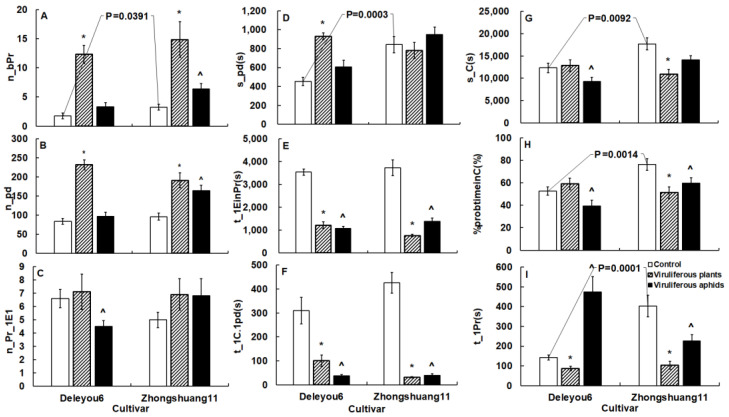
The surface-mesophyll-related variables of *Brevicoryne brassicae* probing behavior on the two oilseed rape cultivars in the TuMV-infected and mock-inoculated treatments. The figure’s values display mean ± standard error (SE). Following the square-root transformation for frequency variables, natural log transformation for time variables, and square-arcsine for percentage variables, these data were compared using Student’s *t*-tests (for Gaussian variables) or Mann–Whitney *U*-tests (for non-Gaussian variables). It was decided to set the significance level at *p* < 0.05. The *p*-value on the white columns indicates a statistical difference between the two cultivars; the * on the gray columns denotes a statistical difference between infected plant and mock-inoculated control in the same cultivar; the ^ on the black columns indicates a statistical difference between viruliferous aphids and non-viruliferous aphids in the same cultivar. (**A**): Number of short probes; (**B**): Number of pd; (**C**): Number of probes before the first E1; (**D**): Total duration of pd; (**E**): Time from the beginning of that probe to the first E; (**F**): Time from the beginning of the first probe to the first pd; (**G**): Total C duration with pd; (**H**): Percentage of probing spent in C; (**I**): Time to the first probe from the start of EPG. The acronyms for the variables are defined in Table S1.

**Figure 4:**
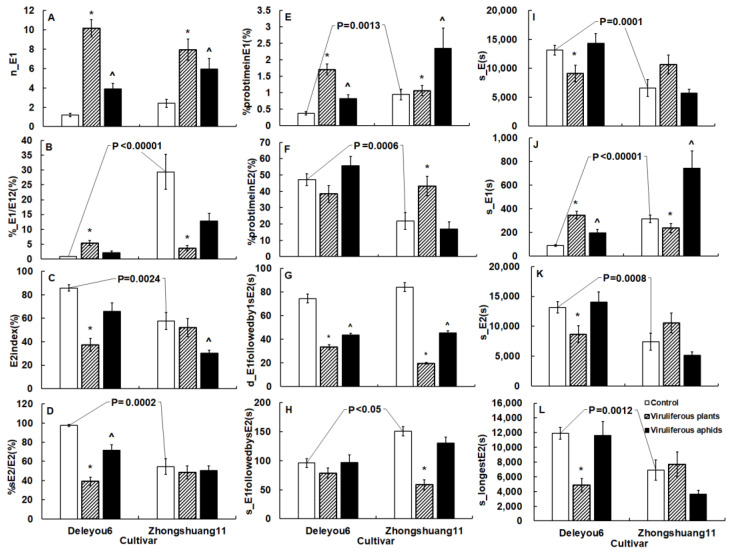
The phloem-related variables of *Brevicoryne brassicae* probing behavior on the two oilseed rape cultivars in the TuMV-infected and mock-inoculated treatments. The figure’s values display mean ± standard error (SE). Following the square-root transformation for frequency variables, natural log transformation for time variables, and square-arcsine for percentage variables, these data were compared using Student’s *t*-tests (for Gaussian variables) or Mann–Whitney *U*-tests (for non-Gaussian variables). It was decided to set the significance level at *p* < 0.05. The *p*-value on the white columns indicates a statistical difference between the two cultivars; the * on the gray columns denotes a statistical difference between infected plant and mock-inoculated control in the same cultivar; the ^ on the black columns indicates a statistical difference between viruliferous aphids and non-viruliferous aphids in the same cultivar. (**A**): Number of E1 periods; (**B**): Relative amount of E1 on E12; (**C**): Percentage of the time of the E2 after the start of the first E2; (**D**): Relative amount of sE2 on E2; (**E**): Percentage of probing spent in E1; (**F**): Percentage of probing spent in E2; (**G**): Duration of the E1 followed by the first sustained E2; (**H**): Total duration of E1 followed by sustained E2; (**I**): Total duration of the E phases; (**J**): Total duration of E1; (**K**): Total duration of E2 periods; (**L**): Duration of the longest E2. The acronyms for the variables are defined in Table S1.

Microscopic and EPG revealed the aphid resistance of two oilseed rape cultivars. At various tissue levels, some key variables with statistical differences reflected resistance (Table 2 and Table 3, Figure 2, Figure 3 and Figure 4).

The anatomical leaf structures of the two oilseed rape cultivars are shown in Figure S1. As shown in Table 2, there were substantial differences in leaf surface properties between the two cultivars. In comparison to ‘Deleyou6’, ‘Zhongshuang11’ showed a thicker upper epidermis (F = 1.90, *p* < 0.05) and more trichomes on the lower surface (F = 3.14, *p* = 0.0033). The length and density of trichomes on the upper surface of the two cultivars were not statistically different (Table 2).

In order to examine aphid resistance between the two cultivars, EPG data from mock-inoculated plants were used as control data because there was no discernible change in aphid feeding behavior between mock-inoculated and uninoculated plants (Table S2). In general, on ‘Deleyou6’, aphids spent considerably less non-probing time (U = 23.00, *p* < 0.00001 for s_np.1E; U = 57.00, *p* = 0.0001 for s_np) and non-phloematic phase (U = 88.00, *p* = 0.0025 for s_nE), and had less probing frequency than on ‘Zhongshuang11’ (Figure 2B–D,G). Other overview variables between the two cultivars showed no significant changes (Figure 2A,E,F).

On the leaf surface of ‘Deleyou6’, aphids began the first probe much earlier than on ‘Zhongshuang11’ (U = 59.00, *p* = 0.0001). Aphids spent less time in the mesophyll of ‘Deleyou6’, produced fewer brief probes, and spent less time on intracellular penetration than in that of ‘Zhongshuang11’. Aphids spent a lot more time in the phloem of ‘Deleyou6’ than ‘Zhongshuang11’, and their metrics for ingestion, including s_E2, s_longestE2, E2index, %sE2/E2, and %probtiminE2, were also significantly greater. However, aphids in the phloem of ‘Deleyou6’ secreted less saliva, as evidenced by s_E1, %_E1/E12, s_E1followedbysE2, and %probtimeinE1 than in the phloem of ‘Zhongshuang11’. Other variables between the two cultivars did not show any significant differences (Table 3).

Based on the results of field observations (Figure 1), the microscopic (Table 2) and EPG data (Table 3), ‘Zhongshuang11’ was classified as an aphid-resistant cultivar with resistance in the epidermis, mesophyll, and phloem, whereas ‘Deleyou6’ was assessed as an aphid-susceptible cultivar.

### 2.4. TuMV Indirectly Modifies Cabbage Aphid Probing Behavior by Infecting Oilseed Rape

TuMV infection in plants significantly changed the probing behavior of cabbage aphids on the two cultivars (Figure 2, Figure 3 and Figure 4).

Aphids on the two TuMV-infected oilseed rape cultivars increased probing frequency and reduced the time before the first phloem contact and first ingestion than on mock-inoculated plants (Figure 2E–G). Other overview variables between the infected and mock-inoculated plants showed no significant changes

In the leaf surface, when compared to mock-inoculated plants of the two cultivars, infected plants’ leaf surfaces were penetrated by aphids earlier (F = 2.24, *p* = 0.0008 for ‘Deleyou6;’ F = 1.51, *p* < 0.05 for ‘Zhongshuang11’) (Figure 3I, Table 3).

Comparing the mesophyll of TuMV-infected plants to mock-inoculated ones, aphids dramatically decreased the pathway time of that probe with the first phloem contact on the two cultivars (Figure 3E). Aphids on infected plants began first cell penetration earlier and greatly increased the frequency of short probes and cell penetration in comparison to mock-inoculated plants (Figure 3A,B,F).

When compared to mock-inoculated plants (F = 1.79, *p* = 0.0021 for s_C; F = 1.32, *p* = 0.0013 for %probtimeinC), aphids significantly reduced the pathway duration and the percentage of pathway duration in relation to the complete probing duration on infected ‘Zhongshuang11’ (Figure 3G,H). These variables did not differ significantly between infected and mock-inoculated ‘Deleyou6’. Aphids stayed in the mesophyll cells for a longer period on infected ‘Deleyou6’ than mock-inoculated plants (U = 18.00, *p* < 0.00001), but there was no significant difference between infected and mock-inoculated ‘Zhongshuang11’ (Figure 3D).

In the phloem, aphids on infected plants of the two cultivars significantly increased salivation frequency but decreased the duration of salivation before the initial sustained ingestion in comparison to mock-inoculated plants (Figure 4A,G, Table 3).

Cabbage aphid probing behavior on TuMV-infected ‘Deleyou6’ differed from that on TuMV-infected ‘Zhongshuang11’.

In the mesophyll and phloem, when compared to mock-inoculated plants, aphids on infected ‘Zhongshuang11’ significantly reduced the pathway duration and the proportion of time for pathway duration (Figure 3G,H, Table 3), required less salivation duration before sustained ingestion (U = 26.00, *p* < 0.00001), and achieved a higher percentage of ingestion (F = 1.17, *p* = 0.0053) (Figure 4F,H, Table 3). These variables did not differ significantly between infected and mock-inoculated ‘Deleyou6’. Aphids on infected ‘Deleyou6’ spent a longer duration of time tasting in the mesophyll cells, spent significantly less time in the phloem, and ingestion (Figure 4C,D,I,K,L, Table 3), but had a significantly higher contribution of salivation phase to complete probing than on mock-inoculated plants (Figure 4E, Table 3). However, there was no significant difference in these variables between infected and mock-inoculated ‘Zhongshuang11’ (Table 3).

Some phloem variables changed in opposite trends by TuMV infection on the two cultivars. Aphids contributed a higher rate of salivation to the phloem phase in the infected phloem of ‘Deleyou6’ than in the phloem of mock-inoculated plants (U = 20.00, *p* < 0.00001), and the other way around held for infected ‘Zhongshuang11’, which showed a lower contribution rate (U = 66.00, *p* = 0.0003) than mock-inoculated plants (Figure 4B). Similarly, aphids spent a longer salivation phrase on infected ‘Deleyou6’ (U = 9.00, *p* < 0.00001) but a shorter length on infected ‘Zhongshuang11’ (F = 2.25, *p* = 0.0376) (Figure 4J, Table 3).

### 2.5. TuMV Directly Modifies Cabbage Aphid Probing Behavior by Infecting Aphids

Cabbage aphid probing behavior was changed by vectoring TuMV on the two cultivars (Figure 2, Figure 3 and Figure 4).

When compared to non-viruliferous aphids on the two cultivars, viruliferous aphids dramatically reduced the time before initial contact with phloem from the EPG start (Figure 2E).

Viruliferous aphids in the mesophyll of the two cultivars took less time to reach the phloem for the first time (U = 0.00, *p* < 0.00001 on ‘Deleyou6;’ F = 1.13, *p* < 0.01 on ‘Zhongshuang11’) and had less pathway phase to complete probing (F = 1.33, *p* = 0.0439 on ‘Deleyou6;’ F = 1.24, *p* = 0.0240 on ‘Zhongshuang11’) than non-viruliferous aphids (Figure 3E,H, Table 3). On the two cultivars, viruliferous aphids penetrated mesophyll cells significantly earlier than non-viruliferous aphids. For example, viruliferous aphids reduced the time by nearly nine times on ‘Deleyou6’ (F = 2.02, *p* < 0.01) and by more than ten times on ‘Zhongshuang11’ (F = 1.03, *p* < 0.01) (Figure 3F, Table 3).

In the phloem of the two cultivars, viruliferous aphids secreted more saliva but spent less time secreting saliva before the initially sustained ingestion than non-viruliferous aphids (Figure 4A,E,G,J, Table 3).

Viruliferous cabbage aphid probing behavior on ‘Deleyou6’ differed from that on ‘Zhongshuang11’.

On ‘Deleyou6’, viruliferous aphids spent more time in non-probing activity before first contact with phloem (1225.04 ± 140.38 s) than non-viruliferous aphids (565.83 ± 17.46 s, U = 21.00, *p* < 0.00001), but spent less time (823.00 ± 139.17 s) than non-viruliferous aphids (1381.80 ± 260.06 s, F = 1.98, *p* = 0.0131) on ‘Zhongshuang11’ (Figure 2D).

In the leaf surface, viruliferous aphids on ‘Deleyou6’ penetrated leaves later than non-viruliferous aphids (U = 30.00, *p* < 0.00001), but earlier on ‘Zhongshuang11’ than non-viruliferous aphids (F = 1.28, *p* = 0.0098) (Figure 3I, Table 3).

In the mesophyll of ‘Deleyou6’, viruliferous aphids reduced probing frequency before first contact with phloem (F = 1.42, *p* = 0.0129) and shortened the pathway time (F = 1.34, *p* = 0.0277) when compared to non-viruliferous aphids. However, compared to non-viruliferous aphids, viruliferous aphids showed no significant differences in these variables on ‘Zhongshuang11’ (Figure 3C,G, Table 3).

On ‘Zhongshuang11’, viruliferous aphids produced more brief probes (F = 1.87, *p* = 0.0377) and more punctures in cells (F = 1.66, *p* = 0.0005) than non-viruliferous aphids. On ‘Deleyou6’, however, viruliferous aphids did not differ significantly from non-viruliferous aphids (Figure 3A,B, Table 3).

In the phloem of ‘Zhongshuang11’, viruliferous aphids produced a lower ingestion index than non-viruliferous aphids (U = 93.00, *p* = 0.0040), but not on ‘Deleyou6’ (Figure 4C, Table 3). Viruliferous aphids had a lower proportion of sustained ingestion to ingestion phase in the phloem of ‘Deleyou6’ than non-viruliferous aphids (U = 89.50, *p* = 0.0029), whereas the percentage was similar in the phloem of ‘Zhongshuang11’ (Figure 4D, Table 3).

## 3. Discussion

### 3.1. Assessment of TuMV-Resistance

In this study, the two cultivars were selected mainly because of the same virus incidence (averaging around 30%) when the two cultivars were compared in the field. Similar to the results from the insect-proof chamber, it was discovered that there was no significant difference in the average TuMV incidence between ‘Deleyou6’ and ‘Zhongshuang11’. Both TuMV-resistance levels were rated as medium resistance even though the average disease index between the two cultivars greatly varied. This matched the results of China’s national crop variety examination and approval. The disease index is calculated by the number of infected plants and the disease infection severity, which is categorized into five levels based on symptoms [29]. Therefore, the resistance level is a qualitative indicator, and most of the resistance levels cover the average disease index within a range of intervals, suggesting that resistance levels may be similar in conditions of different disease indices.

### 3.2. Assessment of Aphid Resistance

An insect’s choice of a plant is a complicated process involving several cues and responses. Aphids probe faster and longer after landing on a preferred host than on a non-preferred host [30]. The number of probe and non-probing phases before the first phloem phase are considered important events in host plant recognition and acceptance [2]. In our study, cabbage aphids on ‘Deleyou6’ spent more time per probe but less time on non-probing and non-phloemic behavior than those on ‘Zhongshuang11’, implying that aphids on ‘Deleyou6’ may spend more time in the phloem. ‘Deleyou6’ appeared to be attractive/suitable to cabbage aphids, whereas ‘Zhongshuang11’ appeared to be obstructive/unsuitable. Attractive and obstructive factors can be found at various levels of the plants, including the leaf surface, mesophyll, and phloem.

After landing on the leaf surface or when reaching the plant by walking, physicochemical characteristics on the plant surface influence insect behavior [31]. These include epicuticular waxes, trichome exudates, texture, topology, odor, and color on the plant surface [31], which are used by plants to resist aphid infestation, and also by aphids to recognize their host plant before making test punctures to find a feeding site [2]. The structural traits of the leaf epidermis may affect the time required for the first probe [32]. Trichomes are the unique structure of epidermal tissue in most plants [33]. Their main function is to resist the invasion of pathogens, mechanically block the movement of aphids on the plant surface, and secrete mucus or toxins to resist aphids [34]. In oilseed rape, ‘Zhongshuang11’ had a thicker upper epidermis and more trichomes on the lower epidermis than ‘Deleyou6’. Aphid probing behavior may be hampered by these leaf features of ‘Zhongshuang11’. In comparison to ‘Deleyou6’, aphids on the leaf upper surface of ‘Zhongshuang11’ delayed the first probe, implying that the thick upper epidermis could be indicative of a resistance factor at the plant surface level. The findings are consistent with those of Hao et al. [28], who found that the relatively thick upper epidermis and bushy, long trichomes of the oilseed rape cultivars ‘Zhongyou821’, ‘Huiyou50’, and ‘Wanyou14’ prevented aphid penetration.

Test punctures play an important role in the recognition process of host plants by insects [35]. The aphid briefly inserts and removes its stylet several times into the plant’s epidermal cells. In the background, the insect exchanges with the host plant, causing a battery of chemical and biochemical reactions, resulting in two possible outcomes: (1) either the insect finds a feeding site and initiates sustained phloem ingestion, in which case there is host plant acceptance; or (2) the insect may encounter resistance that inhibits its ability to feed successfully on the plant on which it has landed [2]. The pathway phase may therefore be extended by any intercellular or intracellular elements in the mesophyll that block the aphid stylet from entering the phloem. In our study, aphids spent much more time in the pathway phase and cell penetration and produced more brief probes on ‘Zhongshuang11’ than on ‘Deleyou6’, implying that there may be intercellular or intracellular obstructive factors in the mesophyll of ‘Zhongshuang11’.

Host plant acceptance is an ultimate and crucial result of aphid host-seeking behavior. Host acceptance or selection occurs when the insect does not encounter or successfully circumvents plant resistance and extends its phloem-feeding period [2]. The E1 (representing salivation) and E2 (indicating ingestion) indices for plant resistance or susceptibility are commonly supplied [36]. Aphids puncture several sieve tube molecules before determining the feeding site, which is often accompanied by prolonged periods of saliva secretion, ranging from 5 s to 30 min, to balance the plant wound response and avoid phloem proteins blocking sieve tube molecules and chemical defense mechanisms [32,37,38]. On ‘Zhongshuang11’, cabbage aphids took significantly longer to salivate than on ‘Deleyou6’. Furthermore, aphids on ‘Zhongshuang11’ released saliva for a longer period of time prior to ingestion and consumed less sap than on ‘Deleyou6’. Aphids on ‘Zhongshuang11’ may encounter plant defenses, feeding inhibitors, and/or insufficient nutrition in the phloem sap [39,40]. Based on the EPG results and characteristics of the leaf surface, we discovered that various factors at multiple distinct leaf tissue levels on ‘Zhongshuang11’, such as surface, epidermis, mesophyll, and phloem, inhibited aphid feeding behavior. Aphids were also substantially more common on ‘Deleyou6’ than on ‘Zhongshuang11’ at all three locations (Chuxiong, Hefei, and Hangzhou), according to the three-year survey’s findings. As a result, we assigned aphid “resistance” to ‘Zhongshuang11’ and “susceptibility” to ‘Deleyou6’.

### 3.3. Indirect Effects of TuMV Infection

Phytoviruses have been demonstrated to use different mechanisms to improve their spread, including manipulating their vector’s activity and transmission efficiency directly or through their shared host plants [2]. Vector activity is related to a set of behaviors on which the phytovirus relies to reach new hosts. It includes host-seeking behavior, probing and feeding behavior, and dispersal (or shifting) behavior [2].

Viral infections are known to cause a range of physiological and metabolic changes in the host plant, which are observed to influence the behavior and fitness of insect vectors as well [11]. Aphids use an array of visual, volatile, and gustatory cues to find phytovirus-infected plants [11]. Plants infected with a virus are preferred by the insect vector over healthy plants. This preference/early attraction is related to a combination of disease symptomatology (for example, yellowing of the leaves attracts insects) and changed volatile emission spectra [41]. Visual cues emanating from virus-infected plants make the plants more apparent to their vectors [5]. After TuMV infection of brassica crops, the veins in the plant’ s new leaves became increasingly obvious and gradually turned into mottled leaves, and the leaves shrank and grew slowly [25]. However, the occurrence of TuMV infection hardly correlates with leaf color [42]. Regarding odor cues, it has also been shown that the plant volatile organic compounds (VOCs) are qualitatively and/or quantitatively modified following phytovirus infection [2]. Bak et al. also found that ethylene-induced changes in volatile production may mediate aphid attraction to infected plants, as choice assays were conducted in the dark, and thus, no visual cues were available [43]. In our study, aphids penetrated leaves more quickly on oilseed rape with TuMV infection than on mock-inoculated plants because the TuMV infection lessened the obstacle or increased their attraction to aphids at the leaf surface level. Similarly, green peach aphids preferred tobacco infected with TuMV and had higher fecundity on tobacco and *A. thaliana* infected with TuMV [44]. The plants, after being inoculated with TuMV, could release VOCs which may attract the aphids to feed the plants and transmit the TuMV to new plants [5,25]. In a study of the interaction between TuMV-transmitting aphids and brassica crops, brassica crops produce some chemical volatiles to regulate the behavior of aphids. Sesquiterpenes and monoterpenes, including (E)-α-vanilene, (E)-β-butene, and camphor, affect the feeding behavior of insects. In volatile metabolome analysis, the VOCs were different after inoculation with TuMV in resistant B80124 and susceptible B80461, and the degree of downregulation of differentially expressed metabolites was more obvious than the degree of upregulation. This was linked to the use of VOCs to attract aphids. The VOC compositions and concentrations were different between inoculated and non-inoculated cultivars in brassica crops, indicating that there were significant differences in gene expression and metabolism [25]. Furthermore, the jasmonate acid (JA) pathway regulates the synthesis and release of terpene volatiles, and virus-encoded proteins can interact directly with the JA pathway to lower host resistance and terpenoid volatile production, thereby attracting vector insects and facilitating virus acquisition [45,46]. This attraction for infected plants by aphid vectors could have important implications for virus spread because the number of plants visited daily is a key factor driving virus outbreaks, according to the model of non-persistent virus transmission [47,48]. Mauck and colleagues [14] noted that despite being less palatable to aphids, squash plants infected with the Fny strain of CMV were initially more attractive to aphids, and this was related to increases in the number of VOCs released by infected plants. They proposed that increased VOC emission acts as a deceptive semiochemical signal to lure aphids towards infected plants, while the unpalatability of the infected plants would repel the insects as soon as they acquired virions during the initial probing of the host’s epidermal tissue.

When the aphids puncture and ingest sap, they produce brief superficial probes and intracellular stylet punctures to gather the gustatory and nutritional signals available in infected plants, and the virus is obtained at the same time. The plant tissue fluid was tested by the chemical receptors at the tip of the maxillary stylet and the parapharyngeal region of the esophagus [25]. Following these punctures, aphids leave behind minute holes that heal quickly and facilitate non-persistent virus transmission [4]. According to our research, aphids on TuMV-infected oilseed rape exhibited more brief probes and intracellular punctures than aphids on mock-inoculated plants. These findings are analogous to those of CMV, another non-persistent virus. CMV also increases the number of brief superficial probes and intracellular punctures, and it has been discovered that the number of penetrating cells in infected plants is positively associated with the efficiency of aphids acquiring viruses [4]. When the short duration of their probes and a high number of intracellular stylet punctures were observed, Collar et al. [49] discovered that *M. persicae* could successfully acquire potato virus Y (PVY). After the final intracellular puncture, PVY-transmitting aphids spent less time in stylet pathway activities, implying that non-persistently-transmitted viruses are easily lost when the probe is extended for a longer period of time following viral acquisition. Cabbage aphids on infected plants considerably decreased the time from the beginning of that probe to the first phloem contact in comparison to mock-inoculated oilseed rape, which may have been the reason for the successful vectoring of viral particles.

After TuMV infection, aphids on infected plants increased salivation frequency by approximately nine times that of mock-inoculated ‘Deleyou6’ and three times that of mock-inoculated ‘Zhongshuang11’, suggesting that TuMV infection leads to increased resistance to aphids and exclusion of aphids from feeding, although a significant decrease in salivation before ingestion (s_E1followedbysE2) was found. The traditional hypothesis also supports that non-persistent virus infection is detrimental to long-term vector colonization, thereby facilitating its propagation [5]. CMV-infected cucurbits attract aphids through changes in VOC emission, but the decreased palatability of these plants subsequently repels them [4,14]. In Arabidopsis, CMV also induces unpalatability, which encourages aphid dispersion [9]. The effects of CMV on cucurbits and Arabidopsis probably drive the spread of this non-persistently transmitted virus since CMV acquisition and inoculation are favored by brief probe-feeds by aphids, not by prolonged ingestion [50]. Thus, attraction leading to short-term probing by vector on a non-persistent virus infected plant and rapid dispersal thereafter is considered ideal for non-persistent virus spread [5].

### 3.4. Direct Effects of TuMV Infection

Previous studies have shown that there were significant differences in the drive-ability of non-viruliferous vector insects and viruliferous vector insects to healthy plants and susceptible plants and that non-viruliferous vector insects tend to feed on susceptible plants, while viruliferous vector insects tend to harm healthy plants [51,52]. Furthermore, the predilection only appears at the beginning of the viruliferous aphid-plant interaction. This form of vector preference has been termed conditional preference and is the subject of much current research based on the premise that it represents virus manipulation of the plant and vector [20].

In our investigation, viruliferous aphids secreted more saliva and ingested less sap than non-viruliferous ones. Similarly, the average feeding time of *Bemisia tabaci* Gennadius (Homoptera: Aleyrodidae)*,* the vector of tomato yellow leaf curl China virus (TYLCCNV) on cotton, was shown to be around one-third of that of non-viruliferous whiteflies by He et al. [53], who concluded that while the virus damaged whitefly feeding behavior, this change in behavior facilitated viral transmission. Using EPG, Wan et al. [54] discovered that rice stripe virus (RSV) infection in eclosion *Laodelphax striatellus* Fallén (Homoptera: Delphacidae) males decreased the total time of phloem nutrient feeding but increased the total time of saliva secretion during feeding. Since short- and long-ingestion probes are known to harm plant tissues, they are unlikely to aid in the spread of viruses [55]. Viral infection may, therefore, directly change a vector’s predilection for probing, but it may not occur in a way that favors feeding.

### 3.5. Cultivar Effects of TuMV Transmission

Cultivars also have an impact on indirect and direct modification. After TuMV infecting plants, aphids on infected ‘Deleyou6’ plants had much longer intracellular penetration lengths than on mock-inoculated plants, whereas aphids on infected ‘Zhongshuang11’ plants did not. Increased attraction in mesophyll cells aided aphid acquisition and carrying of non-persistent virus particles, implying that TuMV infection in plants alters aphid probing behavior to aid virus acquisition and that aphids may find it easier to obtain virus particles on the susceptible cultivar ‘Deleyou6’ than on the resistant cultivar ‘Zhongshuang11’.

In comparison to mock-inoculated plants, aphids significantly increased the duration and proportion of salivation on infected ‘Deleyou6’. This indicated that the virus might increase the phloem’s resistance to aphids, hence reducing the amount of aphid feeding. However, compared to mock-inoculated plants, aphids on infected ‘Zhongshuang11’ had decreased salivation (s_E1 and %_E1/E12), which might be related to the high aphid resistance of ‘Zhongshuang11’.

In addition, cabbage aphids on infected ‘Deleyou6’ plants spent less time in the phloem and had a shorter ingestion phase than on mock-inoculated plants. Such a result is consistent with the conventional hypothesis, which highlights the detrimental effects of long-term settling of vectors on non-persistent virus spread [5]. Similarly, measurements of growth parameters carried out on *M. persicae* settling on a PVY-infected tobacco plant revealed a significant decrease in body and head width and body and cornicle length [56]. Delayed body growth and prolonged development duration were reported for *A. gossypii* and *M. persicae*, respectively, on CMV-infected plants [4,9]. The reproduction rate and population growth rate of *A. idaei* were also negatively impacted on *R. idaeus* infected with RLMV or BRNV [6]. These indicate that non-persistent virus infection reduces the quality of the host plant. Another strategy used by non-persistent and non-circulative phytoviruses is the biosynthesis of toxins and feeding deterrents, such as 4MI3M, one aphid feeding-deterrent synthesized by CMV to discourage prolonged sap ingestion by vectors [9]. This resulted in a reduction in the *M. persicae* growth rate [2] because long-feeding probes or phloem-feeding reduce transmission efficiency [57]. Deceptive signaling or attraction leading to short-term probing by vector on a non-persistent virus infected plant and rapid dispersal thereafter is considered ideal for non-persistent virus spread [5].

Unlike the aphid-susceptible cultivars, as an aphid-resistant cultivar, the phloem of ‘Zhongshuang11’ itself owns barriers to aphid feeding. When compared to mock-inoculated plants, cabbage aphids on infected ‘Zhongshuang11’ showed a higher percentage of ingestion-which could be due to the shortened pathway (s_C) and salivation duration (s_E1)-as well as no significant changes in non-probing duration (s_np) during the 6-h interval. TuMV infection, however, may not further limit aphid ingestion due to the strong sap deterrence in the uninfected phloem of ‘Zhongshuang11’. Instead, it may diminish the inhibited degree of feeding, attracting aphids to obtain and transmit the virus. For example, infection with TuMV increases *M. persicae* attraction to and reproduction on infected plants [44,58]. TuMV and NIa-Pro are demonstrated to increase aphid reproduction on infected plants by inhibiting plant defenses [59]. In *Nicotiana benthamiana* L., TuMV NIa-Pro protein was reported to alter the physiology of plants by suppressing callose deposition and increasing the abundance of free amino acids leading to increased *M. persicae* arrestment and reproduction on TuMV infected plants [44].

According to Carmo-Sousa et al. [4], the general rule that a rise in the frequency of brief superficial probes and intracellular punctures followed by a phloem-feeding discouragement enhances virus transmission efficiency may apply to viruses spread in a non-persistent manner. In our study, we saw longer intracellular penetration times and less ingestion on infected ‘Deleyou6’, but not on infected ‘Zhongshuang11’. Thus, the rule was verified on aphid-susceptible cultivars in our study but not on aphid-resistant cultivars. Jing et al. [60] examined the acquisition rate of the small brown planthopper *L. striatellus* for RSV on two rice cultivars, ‘Yandao8’ (planthopper-resistant cultivar) and ‘Wuyujing3’ (planthopper-susceptible cultivar). They discovered that the virus acquisition rate for planthoppers on infected ‘Yandao8’ was 12.0% within 12 h, compared to 42.3% for infected ‘Wuyujing3’, demonstrating that planthopper-resistant cultivars hindered viral acquisition for small brown planthopper. It could be speculated that aphids have easier access to viruses on aphid-susceptible cultivars than on aphid-resistant cultivars.

In contrast to non-viruliferous aphids, viruliferous aphids spent much less time on ‘Deleyou6’ before the first phloem contact and much more time non-probing. This indicates that viruliferous aphids shorten the time it takes to travel from the epidermis to the first phloem contact. However, viruliferous aphids outlasted non-viruliferous aphids in terms of total non-probing duration on ‘Deleyou6’ and non-phloemic duration on ‘Zhongshuang11’, indicating that the attraction of mock-inoculated plants to viruliferous aphids waned as recording progressed.

Viruliferous aphids also respond differently from non-viruliferous aphids to host plant volatiles or other plant characteristics, according to Werner [61] and Medina-Ortega et al. [62]. For instance, viruliferous *M. persicae* is less prone to host VOC than non-viruliferous *M. persicae* [61]. Similarly, compared to non-viruliferous aphids, viruliferous aphids delayed first probing on ‘Deleyou6’, but started earlier on ‘Zhongshuang11’. Although the difference in attraction was attributed to the thickness of the upper epidermis in the assessment of aphid resistance, the differences in VOCs are speculated to be a more important reason for the resistance difference. More research on the differences in VOCs between the two cultivars is required.

The acquisition and subsequent inoculation of non-persistent viruses are positively correlated with short probes and intracellular stylet punctures because aphids must release viruses to live cells in order for viral particles to survive [58,63]. Wang et al. [64] discovered that CMV inoculation efficiency was adversely linked with fasting duration in viruliferous cotton aphids because fasting treatment dramatically reduced the durations of sub-phase pd II-1 and pd II-2. Further investigation revealed that the duration of pd II-2 was most likely a behavioral element related to virus inoculation. In our study, viruliferous aphids started penetrating cells earlier and produced more brief probes and punctures in the cells on ‘Zhongshuang11’ than non-viruliferous aphids, which could be conducive to viral inoculation. Furthermore, viruliferous aphids reduced pathway duration and decreased probing frequency on ‘Deleyou6’ before the initial phloem contact, implying that viruliferous aphids prefer mock-inoculated plants over non-viruliferous aphids.

According to Alvarez et al. [65], the partial plant resistance identified in cultivars ‘Kardal’ and *Solanum stoloniferum* may be effective in restricting the spread of phloem-restricted viruses, such as potato leaf roll virus. Surface-resistance factors, combined with mesophyll- and phloem-localized resistance factors, should reduce the likelihood of being inoculated by persistently transmitted viruses. According to Jing et al. [60], the non-probing period of viruliferous small brown planthoppers on healthy ‘Yandao8’ (planthopper-resistant cultivar) was 0.34 times longer than on ‘Wuyujing3’ (planthopper-susceptible cultivar). After 12 h of feeding on healthy ‘Yandao8’, viruliferous small brown planthoppers were able to transmit the virus, with a transmission rate of 63.3%, which was lower than that (100%) of ‘Wuyujing3’. Small brown planthoppers were discovered to be unsuitable for virus transmission on ‘Yandao8’. However, in our study, these resistant factors from ‘Zhongshuang11’ appeared to have a lower likelihood of acquisition, but not of inoculation, for non-persistently transmitted viruses such as TuMV. This may be related to the different types of insect vectors. Insects vectoring non-persistent viruses can overcome the insect resistance of plants, which could explain the identical TuMV incidence in the field between the two cultivars.

## 4. Materials and Methods

### 4.1. Biomaterials

Cabbage aphids, *Brevicoryne brassicae*, were obtained from oilseed rape plants growing in the greenhouse at the Institute of Vegetables, Zhejiang Academy of Agricultural Sciences, Hangzhou, China (30°22′13.44″ N, 120°21′24.8″ E). The aphids were raised for one year on a cultivar of *Brassica oleracea* var. *capitata* (L.) (Capparales: Brassicaceae) at 25 ± 1 °C, 75 ± 5% relative humidity (RH), and 16:8 (L:D) photoperiod. In a (60 × 60 × 60 cm) rearing cage, 20 apterous aphids were cultured on a potted *B. oleracea* plant. After 10 d, a new *B. oleracea* plant was placed in a new rearing cage, and 20 newly molted apterous aphids were picked and placed on the plant for succession rearing. A single virginoparous apterous aphid was placed on a new *B. oleracea* plant before the experiment started to obtain offspring aphids. Each of this study’s aphids was the descendent of a single virginoparous apterous aphid. The EPG test was performed using newly molted (2 days old) apterous adults.

Two *Brassica napus* var. *napus* (L.) cultivars, namely ‘Deleyou6’ and ‘Zhongshuang11’, were chosen from the collection maintained at the Laboratory of Plant Breeding of Anhui Academy of Agricultural Sciences, China (31°53′40.056″ N, 117°15′5.58″ E). In plastic pots with a 13 cm diameter, plants were cultivated using a mixture of peat moss, vermiculite, organic fertilizer (N + P_2_O_5_ + K_2_O 2%, organic matter 40%, Zhongnuo, Huaian, China), and perlite (10:10:10:1 ratio). The plants were cultivated at a temperature of 25 ± 1 °C, a relative humidity of 75 ± 5% RH, and a photoperiod of 12:12 (L:D) without the use of any additional fertilizer [27,66].

TuMV-CR1, a moderate strain, is the most prevalent line in China [67]. The TuMV isolate CR1 was originally provided by the Tobacco Research Institute, Anhui Academy of Agricultural Sciences, China. When *Brassica rapa* L. ssp. *pekinensis* plants were at the two-leaf stage in a controlled growth chamber at 25 ± 1 °C, 75 ± 5% relative humidity (RH), and 16:8 (L:D) photoperiod. They were inoculated with 5 cm^2^ of frozen stock tissue infected with TuMV-CR1 (stored at −80 °C). A single mechanical inoculation event for TuMV-CR1 produced common stocks of immature, highly symptomatic tissue preserved at −80 °C, which were used for all inoculations. The disease strain was harvested 26 days after inoculation as a virulent source [68].

### 4.2. Field Investigation of Aphid and TuMV Incidence

Two oilseed rape cultivars ‘Deleyou6’ and ‘Zhongshuang11’, were examined in the field for aphid numbers and TuMV incidence over a three-year (2018, 2019, and 2020) in Chuxiong (Yunnan Province, 25°19′12″ N, 101°29′24″ E), Hefei (Anhui Province, 31°52′48″ N, 117°14′24.00″ E), and Hangzhou (Zhejiang Province, 30°18′36″ N, 120°11′24″ E). Aphid preference trials were carried out in the plots selected at all three locations in 2016 and 2017, with 100 aphids released artificially [66]. Without any physical separation, a 6 m by 12 m plot was divided into two 6 m × 6 m squares, each of which was planted with a single cultivar in September (50 cm row spacing, 10 cm plant spacing). The cultivation and maintenance practices, such as fertilizing and watering, followed local customs without the use of any chemicals. Upon counting the number of nymphs and adults on the plants at the four-leaf stage, an assessment of the aphid population was made. By randomly selecting 10 plants from each cultivar, the population of aphids (adults and nymphs) was counted. The top leaves of these 10 plants were then taken and delivered to the lab, where a kit was used to confirm the presence of TuMV.

### 4.3. Identification of TuMV-Resistance of Plants by Artificial Inoculation

The method of Hao et al. [69] was used to obtain mock-inoculated oilseed rape plants and infected plants, and viruliferous and non-viruliferous aphids. The presence of TuMV in symptomatic plants and aphids was confirmed using the double antibody sandwich, enzyme-linked immunosorbent assay DAS-ELISA method, which was performed with a TuMV ELISA kit from SenBeiJia Biological Technology Co., Ltd., Nanjing, China. The method was primarily used to assess whether aphids feeding on infected plants could successfully become viruliferous rather than determining whether a single aphid was viruliferous, which was nevertheless determined with the aid of viral verification of the plant after the single aphid feeding. The method was also used to access whether aphids feeding on mock-inoculated plants are viruliferous and whether plants are viruliferous after aphid exposure or after inoculation. According to the accompanying documentation, 0.1 g of plant leaves or 200 aphids were homogenized and centrifuged for around 20 min (1000× *g*) in 100 µL of 0.1 M phosphate buffered saline solution (PBS, pH 7.4). After being collected, 10 µL of the supernatant was added to 40 µL of sample dilution in the sample wells of an ELISA plate. The negative and positive control wells each received 50 µL of the negative control reagent and 50 µL of the positive control reagent, respectively. The ELISA plate was sealed with parafilm and then incubated at 37 °C for 30 min. The liquid was discarded after the sealing film was taken off. Each well was filled with the washing solution, which was discarded after 30 s. To each well, 50 µL of the enzyme standard reagent was applied. The plate was incubated and washed again, as shown above. Each well received 50 µL of chromogenic agent A, followed by 50 µL of chromogenic agent B, which were both added to and thoroughly mixed. The reaction was terminated after a 10 min incubation at 37 °C upon the addition of 50 µL/well of the termination solution. A microtiter plate reader (Epoch 2; BioTek Instruments, Inc., Winooski, Vermont, USA) was used to read the plates at 450 nm with a zero blank. The critical value was calculated by adding 0.15 to the mean optical density (OD) value of the negative control wells. TuMV was not present in the samples with OD values below the critical value, while the samples with OD values above the critical value tested positive for TuMV.

The approaches of Chen et al. [29] and Hao et al. [69] were used to determine the disease resistance of two oilseed rape cultivars. The five levels of resistance (susceptibility) based on the average disease index (ADI) are immunity: 0; high resistance: 0.1–10.0; medium resistance: 10.1–30.0; medium susceptibility: 30.1–50; high susceptibility: over 50. When selecting cultivars for breeding, cultivars are selected based on disease resistance rather than susceptibility to diseases [30]. In general, a score >50% indicates that the cultivar is susceptible to the virus, resulting in serious yield losses and unsuitability for use in production. Thus, there is no need to differentiate the degree of susceptibility further. However, scores <50% were classified into different scores to determine the TuMV-resistant level of plants [29].

### 4.4. Microscopic Observation of Leaf Surface

The leaf surface feature was observed and investigated following Yan and Wang [37] and Hao et al. [69] methodologies.

### 4.5. EPG Experiments

The EPG technique described by Hao et al. [69] was employed to track apterous adult aphids’ penetration activities on oilseed rape plants. In a Faraday cage, each cultivar was subjected to three treatments. Non-viruliferous aphids on mock-inoculated plants were present in Treatment I (the negative control). Non-virulent aphids on infected plants were used as part of Treatment II. Viruliferous aphids on mock-inoculated plants were used in Treatment III. Within 30 min of being removed from the raising plant, the tethered aphid was rapidly put on the upper side of the mature leaf midrib of the test plant. Each treatment was recorded over thirty times in a laboratory setting with constant lighting and a temperature of 25 ± 1 °C. For each recording, aphids and plants were used only once. For each cultivar, three treatments with over 30 replicates in each treatment, and one aphid and one plant each replicate-i.e., 90 aphids and 90 plants in total-were used. The feeding behavior of *B. brassicae* was monitored for 6 h. The waveform definitions recorded in EPG analyses for each aphid are listed in Table S1.

### 4.6. Statistical Analysis

Depending on data distribution, data were transformed (square-root transformation for the number of occurrences, natural log transformation for the duration, and square-root arcsine transformation for proportion) and analyzed using Mann–Whitney *U*-tests (for non-Gaussian variables) or Student’s *t*-tests (for Gaussian variables) in SAS 9.2 software (SAS Institute Inc., Cary, NC, USA) [69]. The significance level was chosen at *p* = 0.05. The tables and figures that follow only display the variables that are the most important, despite the fact that all statistics were computed on all variables.

## 5. Conclusions

Cabbage aphids on TuMV-infected oilseed rape significantly reduced the time required to first probe and pathway duration, while increasing brief probes and cell penetration, indicating that virus-infected plants were more attractive to aphids at the epidermis and mesophyll levels where the viruses could be acquired more effectively. Viruliferous aphids on mock-inoculated plants reduced pathway duration, increased salivation, and decreased ingestion, suggesting that viruliferous aphids were attracted to mock-inoculated plants and efficiently inoculated the viruses. TuMV employs a pull-push strategy, which involves pulling the vector aphids to virus-infected plants initially and then pushing them to healthy plants before initiating sustained feeding. Moreover, oilseed rape cultivars can influence the TuMV effect on cabbage aphid feeding behavior. Aphids lengthened the time of cell penetration, increased salivation, and decreased ingestion on the aphid-susceptible cultivar ‘Deleyou6’, but decreased salivation and raised the proportion of ingestion on the aphid-resistant cultivar ‘Zhongshuang11’. It is believed that aphids are more likely to acquire and vector the virus on susceptible cultivars than resistant cultivars. Furthermore, viruliferous aphids were attracted to the leaf surface and, on resistant cultivars, produced more brief probes and cell penetration than on susceptible cultivars. Viruliferous aphids may find it simpler to inoculate the virus on resistant cultivars than on susceptible cultivars, inferring from our research. This could explain why the TuMV incidence did not differ significantly between the two cultivars with different resistance to cabbage aphids. To clarify these intricate linkages, more detailed behavioral, physiological, biochemical, and molecular bioassays, as well as mesocosm research and modeling, are required, which may allow the development of more effective crop protection strategies and be helpful in finding the key links in the prevention and control of TuMV.

## Figures and Tables

**Table 1:** The resistance of two *Brassica napus* cultivars to TuMV.

Cultivar	Average Disease Incidence (%)	Average Disease Index (%)	Resistance Rank
Deleyou6	25.80 ± 2.83	29.48 ± 3.87	medium resistance
Zhongshuang11	20.30 ± 2.98	16.25 ± 3.12 *	medium resistance

The table’s values display mean ± standard error (SE). Following the square-arcsine transformation, these data were compared using Student’s *t*-tests. *p* = 0.05 was chosen as the threshold for significance; the * within a column denotes a statistically significant difference between the two cultivars.

**Table 2:** The resistance of two *Brassica napus* cultivars to TuMV.

Variable	Cultivar	
	Deleyou6	Zhongshuang11
Thickness of upper epidermis (μm)	19.06 ± 0.95	29.75 ± 1.51 *
Trichome length (μm)	824.75 ± 48.73	711.23 ± 28.67
Trichome density on the upper surface	15.33 ± 3.04	14.00 ± 1.15
Trichome density on the lower surface	27.40 ± 2.38	40.50 ± 1.50 *

The table’s values display mean ± standard error (SE). The Student’s *t*-test or Mann–Whitney *U*-tests were used to compare these data. The threshold for significance was established at *p* = 0.05; the * within a row represents a statistically significant difference between the two cultivars.

**Table 3:** The main variables associated with behavior modification of the two oilseed rape cultivars.

Main Variables Associated with Behavior Modification	Cultivar					
	Deleyou6			Zhongshuang11		
	Treatment I	Treatment II	Treatment III	Treatment I	Treatment II	Treatment III
Epidermis						
t_1Pr (s)	142.12 ± 12.01	86.20 ± 12.03	474.58 ± 77.17	402.78 ± 53.98	102.84 ± 19.71	227.37 ± 29.55
Mesophyll						
n_bPr	1.75 ± 0.47	12.30 ± 1.56	——	3.25 ± 0.50	14.85 ± 3.08	6.40 ± 0.91
n_pd	——	231.70 ± 12.53	——	——	191.30 ± 19.77	163.85 ± 14.60
s_pd (s)	453.27 ± 43.70	930.79 ± 36.81	——	841.95 ± 85.15	——	——
t_1C.1pd (s)	——	100.00 ± 24.23	37.49 ± 5.69	——	32.28 ± 2.63	40.01 ± 6.17
t_1EinPr (s)	——	1211.67 ± 143.29	1074.91 ± 86.78	——	754.98 ± 50.53	1383.98 ± 136.41
s_C (s)	12,325.42 ± 1094.15	——	9293.24 ± 944.29	17,707.84 ± 1346.55	10,904.90 ± 1092.13	——
%probtimeinC	52.61 ± 3.76		39.41 ± 5.19	76.27 ± 5.14	51.22 ± 5.30	59.56 ± 4.93
Phloem						
n_E1	——	10.15 ± 0.89	3.90 ± 0.56	——	7.95 ± 1.08	5.95 ± 1.08
s_E1 (s)	88.98 ± 5.85	345.11 ± 32.56	195.45 ± 28.35	314.60 ± 32.10	236.15 ± 38.27	742.74 ± 145.31
%_E1/E12	0.81 ± 0.07	5.36 ± 0.82	——	29.39 ± 5.95	3.62 ± 0.90	——
d_E1followedby1sE2 (s)	——	33.37 ± 2.15	43.72 ± 1.40	——	19.66 ± 0.60	45.42 ± 1.89
s_E1followedbysE2 (s)	95.96 ± 7.50	——	——	150.44 ± 7.88	58.71 ± 8.72	——
%probtimeinE1	0.37 ± 0.05	1.70 ± 0.17	0.82 ± 0.12	0.95 ± 0.16	——	2.35 ± 0.62
s_E2 (s)	13,141.15 ± 952.43	8669.17 ± 1425.51	——	7411.50 ± 1392.52	——	——
s_longestE2 (s)	11,905.99 ± 805.27	4853.35 ± 937.68	——	6906.80 ± 1400.80	——	——
E2index	85.85 ± 2.58	37.26 ± 5.44	——	57.57 ± 7.29	——	30.40 ± 2.40
%sE2/E2	97.44 ± 1.24	39.15 ± 4.48	71.67 ± 5.83	54.63 ± 8.29	——	——
Resistance assessment	Aphid-susceptible	TuMV-medium resistant		Aphid-resistant	TuMV-medium resistant	

The table’s values display means ± standard error (SE). Following the square-root transformation for frequency variables, square-arcsine transformation for percentage variables, and natural log transformation for time variables, these data were compared using Student’s *t*-tests (for Gaussian variables) or Mann–Whitney *U*-tests (for non-Gaussian variables). The significance threshold was established at *p* = 0.05. Non-viruliferous aphids on mock-inoculated plants were used in Treatment I. Non-viruliferous aphids on infected plants were used as part of Treatment II. Viruliferous aphids on mock-inoculated plants were used in Treatment III. —— indicates no statistically significant difference in treatment I between the two cultivars or between treatment I and II or III in a single cultivar. The acronyms for the variables are defined in Table S1.

## Data Availability

The associated data produced for this study can be cited: Zhong-Ping Hao. (2023), “Aphid-resistance of Oilseed Rape (Brassica napus) does not Necessarily Impede Turnip Mosaic Virus Transmission”, Mendeley Data, V1, doi: 10.17632/xbtyv58f2f.1.

## Supplementary Materials

The following supporting information can be downloaded at: https://www.mdpi.com/article/10.3390/plants12101972/s1, Table S1: The definitions of the waveforms scored in the electropenetrography (EPG) analyses; Table S2: The main variables of the *Brevicoryne brassicae* feeding behavior without significant difference between mock-inoculated and uninoculated plants on two oilseed rape cultivars. Figure S1: The anatomical structures of leaves of the two oilseed rape cultivars. The upper-most first leaf of each cultivar was fixed and photographed using semi-thin Sect. (3–4 µm) immediately after EPG recording ended. A Zhongshuang11; B Deleyou6. (Bar = 100 μm).
