# Supplementary material for: Aphids on Aphid-Susceptible Cultivars Have Easy Access to Turnip Mosaic Virus, and Effective Inoculation on Aphid-Resistant Cultivars of Oilseed Rape (Brassica napus)
Source: Plants (Basel). 2023 May 13;12(10):1972. doi: 10.3390/plants12101972 (PMC10221937; doi:10.3390/plants12101972)
Supplement: Supplementary file 1 [file plants-12-01972-s001.zip › plants-2325219-supplementary.pdf]

Supplementary material

Table S1. The definitions of the waveforms scored in the electropenetrography (EPG) analyses.

Acronym	Variable type	Definition
General		
n_Pr	Frequency	Number of probes
s_Pr	Time	Total probing time
s_nE	Time	Total duration of the no phloematic phase
s_np	Time	Total time of the non-probing intervals
s_np.1E	Time	Duration of the nonprobe period before the 1st E
t_1E2rec	Time	Time from the start of EPG to the 1st E2
t_1Erec	Time	Time from the start of EPG to the 1st E
Surface-mesophyll (Leaf)		
t_1Pr	Time	Time to the first probe from the start of EPG
n_bPr	Frequency	Number of short probes (C < 3 minutes)
n_Pr.1E1	Frequency	Number of probes before the 1st E1
t_1C.1pd	Time	Time from the beginning of the 1st probe to the first pd
n_pd	Frequency	Number of pd
s_pd	Time	Total duration of pd
t_1EinPr	Time	Time from the beginning of that probe to the 1st E
s_C	Time	Total C duration with pd
%probtimeinC	Index	% of probing spent in C
Phloem		
s_E	Time	Total duration of the E phases
n_E1	Frequency	Number of E1 periods
s_E1	Time	Total duration of E1

d_E1followedby1sE2	Time	Duration of the E1 followed by the first sustained E2 (> 10 min)
s_E1followedbysE2	Time	Total duration of E1 followed by sustained E2 (> 10 min)
%_E1/E12	Index	Relative amount of E1 on E12
s_E2	Time	Total duration of E2 periods
s_longestE2	Time	Duration of the longest E2
E2index	Index	phloemian index: % of the time of the E2 after the start of the 1st E2
%sE2/E2	Index	Relative amount of sE2 on E2
%probtimeinE1	Index	% of probing spent in E1
%probtimeinE2	Index	% of probing spent in E2

Table S2. The main variables of the *Brevicoryne brassicae* feeding behavior without significant difference between mock-inoculated and uninoculated plants on two oilseed rape cultivars.

Variable[a]	Cultivar			
	Deleyou6		Zhongshuang11	
	Mock-inoculated	uninoculated	Mock-inoculated	uninoculated
Overview				
n_Pr	9.35±1.39	9.40±1.30	14.70±1.82	16.05±1.68
s_Pr(s)	24651.52±868.06	25245.99±858.69	26706.21±300.27	26499.59±330.30
s_np(s)	603.24±9.38	625.99±13.42	1820.34±264.77	2165.59±299.72
s_np.1E(s)	565.83±17.46	572.71±17.20	1381.80±260.0	1348.07±225.3

			6	6
s_nE(s)	11857.36±687.43	11971.27±744.12	18122.14±1584.99	18216.98±1386.48
t_1E2.rec(s)	11060.70±1107.31	12258.04±1166.64	16144.02±1843.19	15401.84±1839.12
t_1Erec(s)	10969.90±1091.69	11124.74±974.30	13198.30±1542.12	13563.89±1274.34
Surface				
t_1Pr(s)	142.12±12.01	166.14±15.74	402.78±53.98	400.73±46.50
Mesophyll				
t_1C.1pd(s)	309.93±55.01	357.67±49.65	426.06±43.59	391.42±54.01
n_bPr	1.75±0.47	1.95±0.39	3.25±0.50	3.20±0.63
n_pd	83.65±7.48	91.25±7.11	96.00±9.32	101.40±10.60
s_pd(s)	453.27±43.70	466.21±47.70	841.95±85.15	880.26±106.77
n_Pr_1E1	6.60±0.69	6.35±0.41	5.00±0.58	6.05±0.67
t_1EinPr(s)	3535.31±133.57	3621.19±156.07	3728.27±341.17	3561.33±404.81
s_C(s)	12325.42±1094.15	12589.79±951.51	17707.84±1346.55	17262.92±1190.56
%probtimeinC(%)	52.61±3.76	52.76±3.22	76.27±5.14	72.50±5.65
Phloem				
s_E(s)	13127.24±834.	13304.47±773.	6582.47±1447.	6624.15±1248.

	05	09	55	00
n_E1	1.20±0.16	1.40±0.17	2.40±0.43	1.85±0.29
s_E1(s)	88.98±5.85	91.43±3.97	314.60±32.10	344.36±38.55
%_E1/E12(%)	0.81±0.07	0.96±0.09	29.39±5.95	29.33±4.92
d_E1followedby1s	74.37±3.77	80.17±3.63	84.06±3.88	81.13±4.29
E2(s)				
s_E1followedbysE	95.96±7.50	97.06±6.46	150.44±7.88	159.31±7.51
2(s)				
%probtimeinE1(%)	0.37±0.05	0.37±0.05	0.95±0.16	1.09±0.17
s_E2(s)	13141.15±952.	13874.04±101	7411.50±1392.	7756.34±1507.
	43	0.31	52	32
s_longestE2(s)	11905.99±805.	12113.24±926.	6906.80±1400.	6294.55±1355.
	27	72	80	10
E2index(%)	85.85±2.58	86.92±2.27	57.57±7.29	58.33±5.71
%sE2/E2(%)	97.44±1.24	97.62±1.36	54.63±8.29	52.72±8.25
%probtimeinE2(%)	47.22±3.70	49.42±3.22	21.79±5.24	19.65±5.26

[a] The values in the table show mean±standard error (SE). The data were compared using the student *t* test (for Gaussian variables) or the Mann–Whitney *U* test (for non-Gaussian variables) after the square-root transformation for frequency variables, natural log transformation for time variables, and square arcsine for percentage variables. The significance level was chosen at $P < 0.05$. The acronym of the variables was defined in Table S1.

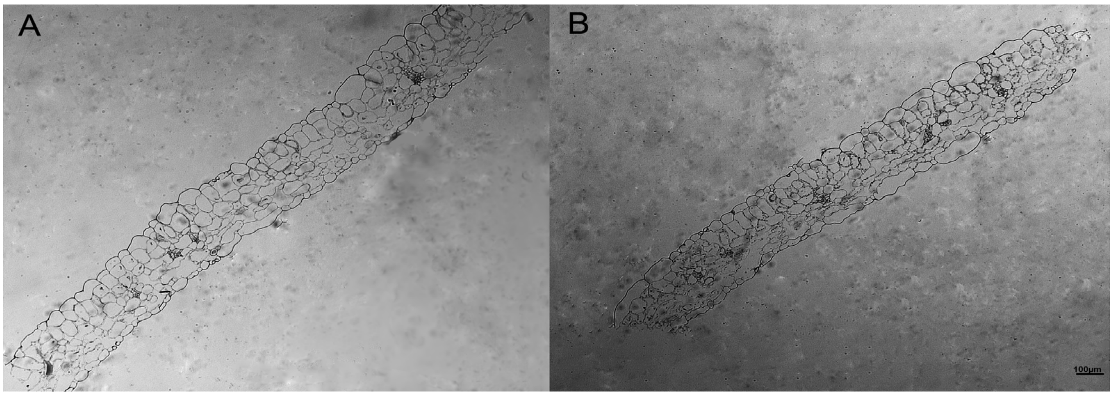

Figure S1. The anatomical structures of leaves of the two oilseed rape cultivars. The upper-most first leaf of each cultivar was fixed and photographed using semi-thin Sect. (3–4 μm) immediately after EPG recording ended. (**A**) Zhongshuang11; (**B**) Deleyou6. (Bar =100 μm).
